# No Contagious Pleuro-pneumonia in Kansas

**Published:** 1895-05

**Authors:** 


Not Pleuro-pneumonia.—The unconfirmed report that several herds of cattle located in eastern Morris County, Kansas, were affected with contagious pleuro-pneumonia was thoroughly investigated yesterday by experts belonging to the United States Bureau of Animal Industry and the Kansas State Live Stock Sanitary Commission. Dr. W. S. Devoe, travelling inspector of the United States Bureau; Albert Dean, the superintendent of the Texas quarantine department of the Bureau west of the Mississippi; Dr. N. S. Mayo, Kansas acting State veterinarian; J. W. Johnson, J. W. Moore, and John T. Brown, who compose the Kansas State Live Stock Sanitary Commission, were among the number.

Daniel Lemay, D.V.S., senior surgeon of the 7th United States cavalry, now stationed at Ft. Riley, for four years beginning with 1880 was State veternarian of Maryland, during which time, with the co-operation of the United States Government, the State was engaged in stamping out contagious pleuro-pneumonia within its confines, and during that time Dr. Lemay had slaughtered hundreds of animals, and, therefore, is thoroughly competent to pass judgment on suspected cases.

The first herd investigated was that of John Furney, four miles southeast of Alta Vista, who had in the feed lots on his farm originally sixty-three head of coming three-year-old stockers that he was roughing through the winter on corn fodder that was made by the hot winds before it had reached maturity, with here and there an abortive nubbin. The herd had no shelter other than the canopy of heaven and an open irregular Osage orange hedge, a kind of substitute for a wind break. The fodder was almost devoid of any nutritive properties, and no grain had been given the cattle. They could not be expected to appear in the best of condition.

About three weeks ago one of the more timid succumbed to the inevitable. The fodder was thoroughly covered with dust, and for sometime an occasional cough was heard in the herd. Others in the surrounding section of country that were roughing through bunches on the same kind of rations reported some cough, and, like unto old witchcraft times, a sentiment grew, and one individual, more nervous than others, got shaky and asked advice of the State Board. Dr. Mayo appeared on the farm of Mr. Furney and suggested that one of the thinnest be slaughtered and a post-mortem be held, which was accordingly done. None of those present at this autopsy had had any personal experience with the supposed disease, and it was thought advisable to report to the State authorities and suggested that the United States Government Bureau be advised in the matter, which was accordingly done.

In the meantime between the zeal of the ever over-zealous newspaper man and the unintentional indiscretion of State-house officials, it was flashed by wire all over the United States and cabled to Europe, that Kansas herds were affected with contagious pleuro-pneumonia. Secretary Morton, of Washington, and Dr. Salmon, chief of the United States Bureau at once ordered Dr. Devoe to proceed and make a thorough examination. An animal, a long two year old, the thinnest and most woebegone member of the herd, was killed, and the autopsy showed, as was pronounced by Dr. Devoe, that there were no indications of the dreaded disease. Mr. Furney was much relieved, in common with his neighbors, whose herds had been—like his own—quarantined by the State Board until the question was definitely settled.

Several stockmen extended invitations to members of the investigating body to visit their herds with a view of holding post-mortems. After consulting about the supposed source of the trouble they went to J. W. Bond's farm, which lay about one mile southwest. He had forty-five head of coming three-year-olds that he had been in possession of for eighteen months. that run in feed lots sheltered by a grove of second growth size timber, and had been fed the same ration of hot-wind-cured corn fodder. There was some little in the favor of Mr. Bond's fodder, as a major portion of his corn grew on the creek bottom and first bench land, and was when the hot winds struck it a little more near maturity. The herd was in better condition generally than was that of Mr. Furney, save that one unfortunate coming three-year-old had run down and about four weeks ago one of his hock-joints enlarged so that he could not well get around in the rustle for forage. Of course he was the most symptomatic individual, hence was selected as the victim for the post-mortem. Dr. Lemay having done the operating on the former animal was again selected to "carve dat 'possum." After a thorough examination of the internal organs it was decided, as was the other case, that there were no indications of the supposed disease, and further that it was useless to proceed with the examination, as Dr. Devoe decided that there were no indications, either external or internal of contagious pleuro-pneumonia. Dr. Lemay stated to the writer that the autopsies showed that the trouble was not contagious pleuro-pneumonia, but from all the symptomatic developments it was the result of anæmia or an impoverished condition due to the lack of a proper ration. Dr. Devoe said: "You may state for the benefit of the general public that after a thorough investigation there is no evidence, either historical or symptomatic, that the trouble is contagious pleuro-pneumonia." And thus ended the investigation.—*Kansas City Telegram.*

